# *POU6F2* mutation in humans with pubertal failure alters GnRH transcript expression

**DOI:** 10.3389/fendo.2023.1203542

**Published:** 2023-08-01

**Authors:** Hyun-Ju Cho, Fatih Gurbuz, Maria Stamou, Leman Damla Kotan, Stephen Matthew Farmer, Sule Can, Miranda Faith Tompkins, Jamala Mammadova, S. Ayca Altincik, Cumali Gokce, Gonul Catli, Fuat Bugrul, Keenan Bartlett, Ihsan Turan, Ravikumar Balasubramanian, Bilgin Yuksel, Stephanie B. Seminara, Susan Wray, A. Kemal Topaloglu

**Affiliations:** ^1^ Cellular and Developmental Neurobiology Section, National Institute of Neurologic Disorders and Stroke, National Institutes of Health, Bethesda, MD, United States; ^2^ Division of Pediatric Endocrinology, Faculty of Medicine, Cukurova University, Adana, Türkiye; ^3^ Harvard Reproductive Sciences Center, The Reproductive Endocrine Unit and The Endocrine Unit of the Department of Medicine, Massachusetts General Hospital, Boston, MA, United States; ^4^ Division of Pediatric Endocrinology, İzmir Tepecik Training and Research Hospital, Health Sciences University, İzmir, Türkiye; ^5^ Division of Pediatric Endocrinology, Faculty of Medicine, Ondokuz Mayis University, Samsun, Türkiye; ^6^ Division of Pediatric Endocrinology, Faculty of Medicine, Pamukkale University, Denizli, Türkiye; ^7^ Division of Endocrinology, Faculty of Medicine, Mustafa Kemal University, Hatay, Türkiye; ^8^ Division of Pediatric Endocrinology, Faculty of Medicine, Selcuk University, Konya, Türkiye; ^9^ Department of Pediatrics, Division of Pediatric Endocrinology, University of Mississippi Medical Center, Jackson, MS, United States; ^10^ Division of Pediatric Endocrinology, Massachusetts General Hospital for Children and Harvard Medical School, Boston, MS, United States

**Keywords:** idiopathic hypogonadotropic hypogonadism, GnRH, POU6f2 isoform1, transcription, puberty

## Abstract

Idiopathic hypogonadotropic hypogonadism (IHH) is characterized by the absence of pubertal development and subsequent impaired fertility often due to gonadotropin-releasing hormone (GnRH) deficits. Exome sequencing of two independent cohorts of IHH patients identified 12 rare missense variants in *POU6F2* in 15 patients. *POU6F2* encodes two distinct isoforms. In the adult mouse, expression of both isoform1 and isoform2 was detected in the brain, pituitary, and gonads. However, only isoform1 was detected in mouse primary GnRH cells and three immortalized GnRH cell lines, two mouse and one human. To date, the function of isoform2 has been verified as a transcription factor, while the function of isoform1 has been unknown. In the present report, bioinformatics and cell assays on a human-derived GnRH cell line reveal a novel function for isoform1, demonstrating it can act as a transcriptional regulator, decreasing *GNRH1* expression. In addition, the impact of the two most prevalent *POU6F2* variants, identified in five IHH patients, that were located at/or close to the DNA-binding domain was examined. Notably, one of these mutations prevented the repression of GnRH transcripts by isoform1. Normally, GnRH transcription increases as GnRH cells mature as they near migrate into the brain. Augmentation earlier during development can disrupt normal GnRH cell migration, consistent with some POU6F2 variants contributing to the IHH pathogenesis.

## Introduction

Idiopathic hypogonadotropic hypogonadism (IHH) is a rare genetic disorder characterized by complete or partial pubertal failure caused by gonadotropin-releasing hormone (GnRH) deficiency. According to the olfactory function, IHH is divided into two major forms, normal sense of smell (normosmic IHH, nIHH) and inability to smell, anosmia, defined as Kallmann syndrome (KS). Although nearly 50 genes have been reported to be associated with IHH (1, 2), they account for only 50% of all cases indicating that other associated genes remain to be discovered. Delineating new genes involved in the development and/or function of GnRH neurons is relevant for understanding the basis of IHH pathogenesis in humans.

Exome sequencing (ES) of two independent cohorts of IHH patients identified missense variants in *POU6F2.* Most POU family members act as transcriptional regulators, with many of them controlling cell type-specific differentiation pathways (3, 4). In addition, several POU domain-containing gene products modulate the development, expression, and function of GnRH neurons (5–7). To date, few reports examine the function of *POU6F2*. *POU6F2* was originally cloned from human retina and is also known as retina-derived POU-domain factor-1 (8). *POU6F2* plays a role in corneal development and is a potential risk factor for glaucoma in humans (9). POU6F2 has been reported to be expressed also in the developing midbrain (8), pituitary (10), and kidneys (11), and mutations of POU6F2 have been identified in prolactinomas (12) and in a hereditary predisposition to Wilms tumor (nephroblastomas; 13). Notably, POU6F2 has two distinct isoforms, with isoform2 being a transcriptional regulator while the function of isoform1 is unclear. In this communication, we present evidence that POU6F2 isoform1 can function as a transcription factor repressing *GNRH1* expression and that one of the POU6F2 variants identified in IHH patients reduced the transcriptional activity of isoform1, increasing GnRH expression. Together, these data are consistent with mutations in *POU6F2* contributing to the pathogenesis of IHH.

## Materials and methods

Human experimental protocols were approved by either the Ethics Committee of the Cukurova University Faculty of Medicine and the institutional review board of the University of Mississippi Medical Center or the Human Research Committee at the MGH, Boston, MA. All individuals and/or their legal guardians provided written informed consent.

### Patients

Two large cohorts of IHH patients were screened for POU6F2 variants. The Cukurova cohort consisted of 416 IHH patients (nIHH, n = 331 and KS, n = 85) from 357 independent families recruited in Turkey. The Harvard Reproductive Endocrine Sciences Center’s IHH cohort included 677 nIHH and 632 KS patients recruited nationally and internationally. Reproductive phenotypes suggestive of IHH were deemed present if they exhibited at least one of the following IHH-related phenotypes: micropenis or cryptorchidism (boys), absent puberty by age 13 in girls and by age 14 in boys, primary amenorrhea (girls), and/or a biochemical observation of hypogonadotropic hypogonadism. The KS patients additionally had anosmia/hyposmia as determined by self-reporting and/or physical examination by administering culturally appropriate formal or informal smell tests. Other causes of hypogonadotropic hypogonadism were ruled out by pituitary/hypothalamic imaging and additional anterior pituitary hormone testing. All of the patients except for B II-5 was older than age 17 years at the time of the last evaluation. Therefore, their final diagnosis as IHH or CDGP (A II-1) was achieved.

### DNA sequencing and rare variant analyses

DNA samples for ES were prepared as an Illumina sequencing library, and in the second step, the sequencing libraries were enriched for the desired target using the Illumina Exome Enrichment protocol. The captured libraries were sequenced using Illumina HiSeq2000 Sequencer. The reads were mapped against UCSC (https://genome.ucsc.edu/cgi-bin/hgGateway) hg19. The variants in ES data were filtered against population polymorphism databases TR Variome (14) and gnomAD in the Cukurova cohort and against gnomAD in the Harvard cohort to obtain rare sequence variants (RSVs), defined as variants with <0.001 minor allele frequency (MAF). The resulting RSVs were then screened for variants in *POU6F2* (NM_007252). The presence and segregation of significant variants within pedigrees were verified by Sanger sequencing on an Applied Biosystems PRISM 3130 auto sequencer. ES data were also screened for potentially significant variants in known IHH-associated genes (15).

### Expression of *Pou6f2* isoforms

All animal procedures were approved by NINDS Animal Care and Use Committee and performed in accordance with NIH guidelines. Total RNA was extracted from adult mouse brain, pituitary, testis, and ovaries using TRIzol reagent (Invitrogen, 15596-026) according to the manufacturer’s instructions. Total RNA (1 µg) was used for cDNA synthesis with oligo(dT)_16_ primer and SuperScript III Reverse Transcriptase (Invitrogen, 18080-044) following the manufacturer’s protocol. cDNAs generated with Superscript III or IV from primary GnRH cells maintained in explants for 7–9 days (16; GnRH cells removed from explants at this time show many characteristics of GnRH cells examined in brain slices of adult animals; 17) and two mouse GnRH cells lines (18, 19) were also analyzed. PCR analysis was performed using specific primers on *Pou6f2* exon 8 (forward, 5’-ACACAGACTCAGGTGGGACAA-3’) and exon 9 (reverse, 5’-TTCCCGGTCGTAGTTTAG-CTT-3’) or isoform2-specific primers (forward, 5’-GCCATCTGCAGGTTTGAAA-3’; reverse, 5’-CGTGTTGCTTTAAGCGTTTG-3’) and products compared on 2% agarose gels. cDNA from a human GnRH cell line (20, 21) was made as described above, and human brain cDNA was purchase from GenScript (Piscataway, NJ). PCR analysis for isoform1 and isoform2 was performed using *Pou6f2* exon 10 PRIMERS (forward, 5’-GGACAGGCTCTCAGTGCTAC-3’) and exon 11 (reverse, 5’-AACTCGGTCAGGTTCTGCAT-3’) or *Pou6f2* exon9 primers (forward, 5’-CAGCCTCCCAAGGCAACCTTCTGC-3’) and exon 11 (reverse, 5’-TCAGGGCTTGCCTCT-TATTG-3’). Both sets of primers see isoform1 and isoform2 (primer set 1: isoform1 = 281 bp, isoform2 = 173 bp; primer set 2: isoform1 = 721 bp, isoform2 = 613 bp). Products generated using the first set of primers were subsequently used as template for a nested PCR using isoform2-specific primers (126 bp; forward, 5’-GCCATCTGCAGGTTTGAAAAG-3’; reverse, 5’-AACTCGGTCAGGTTCTGCAT-3’). Products were compared on 1.5%–2% agarose gels.

### Molecular modeling

POU6F2 isoform1 and isoform2 were generated using C-I-TASSER (22) from their amino acid sequences (UniProtKB codes: P78424-1 and P78424-2). Three-dimensional DNA structures were produced using w3DNA (23). POU6F2-DNA docking was simulated by HDOCK using template-free docking settings for the OCT1 DNA-binding site (24, 25). As predicted, POU6F2 isoform1 did not bind to this site. Since isoform1 was previously reported to bind to *Fshβ (* 10), we tested isoform1 against the *Fshβ*-protected site (5’-ATAAGCTTAAT-3’) and separately against an aligned site in the proximal promoter region of *GNRH1* (5’-AAAAGCATAGT-3’). Mutant proteins and folding free energy values for both isoforms were calculated by DynaMut (26). Natural protein flexibility was detected using CABS-flex dynamics (27). Wild-type (WT) vs. mutant protein-DNA-binding free energy values were predicted by SAMPDI (28). All models were rendered using PyMOL molecular graphics software.

### *In vitro* assay for isoform1 variants

Since only isoform1 was found in GnRH cells, an *in vitro* assay for changes in *GNRH1* expression was performed using a human GnRH cell line, FNC-B4-hTERT. FNC-B4 cells were first isolated from fetal olfactory neuroepithelium (20). Telomerase-mediated immortalization was performed on these cells, and the human GnRH cell line (FNC-B4-hTERT) was established (21). Cells were grown in monolayer (37°C, 5% CO_2_) in F-12 Coon’s modification medium (Sigma, F6636) supplemented with penicillin-streptomycin (Gibco, 15140-122) and 10% fetal bovine serum (Sigma, F7524). FNC-B4-hTERT cells were seeded into six-well plates and cultured until ~80% confluency. Cells were then transfected with mock [pcDNA-3.1(+)IRES-GFP], WT-POU6F2-isoform1 (WT), or one of the two isoform1 mutant plasmids (MT1, MT2) using FuGENE^®^ HD (Promega, E2311) following the manufacturer’s instructions. Transfection efficiency was examined in cells 32 h after transfection. The coverslips were fixed, and GFP-labeled cells/defined area was determined. Conditions were determined such that ~50% of the cells were GFP-positive in each group. Experimental groups were then transfected, and 32 h after transfection, culture media were changed to serum-free media for 16 h prior to GnRH stimulation (20). Cells were treated with GnRH (0.2 µM, [D-Trp^6^]-LH-RH, Sigma, L9761) for 3 h and harvested for RNA preparation. Experiments were performed in triplicate. Total RNA was extracted using TRIzol reagent. Here, 250 ng of total RNA of each group was reverse-transcribed into cDNA using 50 µM oligo(dT)_20_ and SuperScript III reverse transcriptase. All cDNA was stored at -20°CC until analysis of GnRH transcript levels using RT-qPCR. qPCR was performed with primers specific for human Beta-Actin (*ACTB;* forward: 5’-CACCATTGGCAATGAGCGGTTC-3’; reverse, 5’-AGGTCT-TTGCGGATGTCCACGT-3’) and GnRH (*GnRH1*; forward, 5’-CAACGCTTCGAATGCACCA-3’; reverse, 5’-ATGTGCAACTTGGTGTAAGGATT-3’). The primer efficiency of Beta-Actin was 92.3% with an R^2^ = 0.9997. The primer efficiency of GnRH was 126.71% with an R^2^ = 0.9877, falling within a “good” efficiency and amplification factor for qPCR (29). qPCR was performed using SsoAdvanced Universal SYBR^®^ Green Supermix (BioRad, 1725271) and StepOne Real-Time PCR System (Applied Biosystems). Samples amplified with the Beta-Actin primers were diluted 1:100. Samples amplified with GnRH primers were diluted 2:3. Each sample was run in triplicate. Each group was run together on the qPCR machine that resulted in three unique runs. The average of all of the automatic thresholds was taken and used to set a manual threshold. ΔΔCт was calculated to compare GnRH expression across treatment groups. This was done by first calculating the mean of the technical triplicates for each sample for each primer. The ΔCт was then calculated by taking the mean Cт value for GnRH and subtracting the mean Cт value for Beta-Actin for each sample. The ΔΔCт was calculated by subtracting the reference treatment condition (Mock) ΔCт from each of the ΔCт of the treatment conditions (WT, MT1, and MT2). Lastly, the relative expression of GnRH in each group was determined by taking 2 to the power of the negative ΔΔCт. In addition, MT1 and MT2 reside in exon 11 known for containing splicing events that distinguish isoform1 and isoform2. As such, the cDNAs from the same experimental groups assayed by qPCR were examined for POU6F2 isoform1 and isoform2 expression using standard PCR methods as described above.

### Statistical analysis

Data are expressed as mean ± SEM, and statistical evaluation was performed using unpaired t-tests (Prism for macOS, v9.3.1). For qPCR, the Mock ΔΔCт was set to 1 and the remaining treatment conditions were adjusted accordingly to compare across experimental runs. Statistical significance between groups was compared using unpaired t-tests across biological triplicates.

## Results

**Figure 1:**
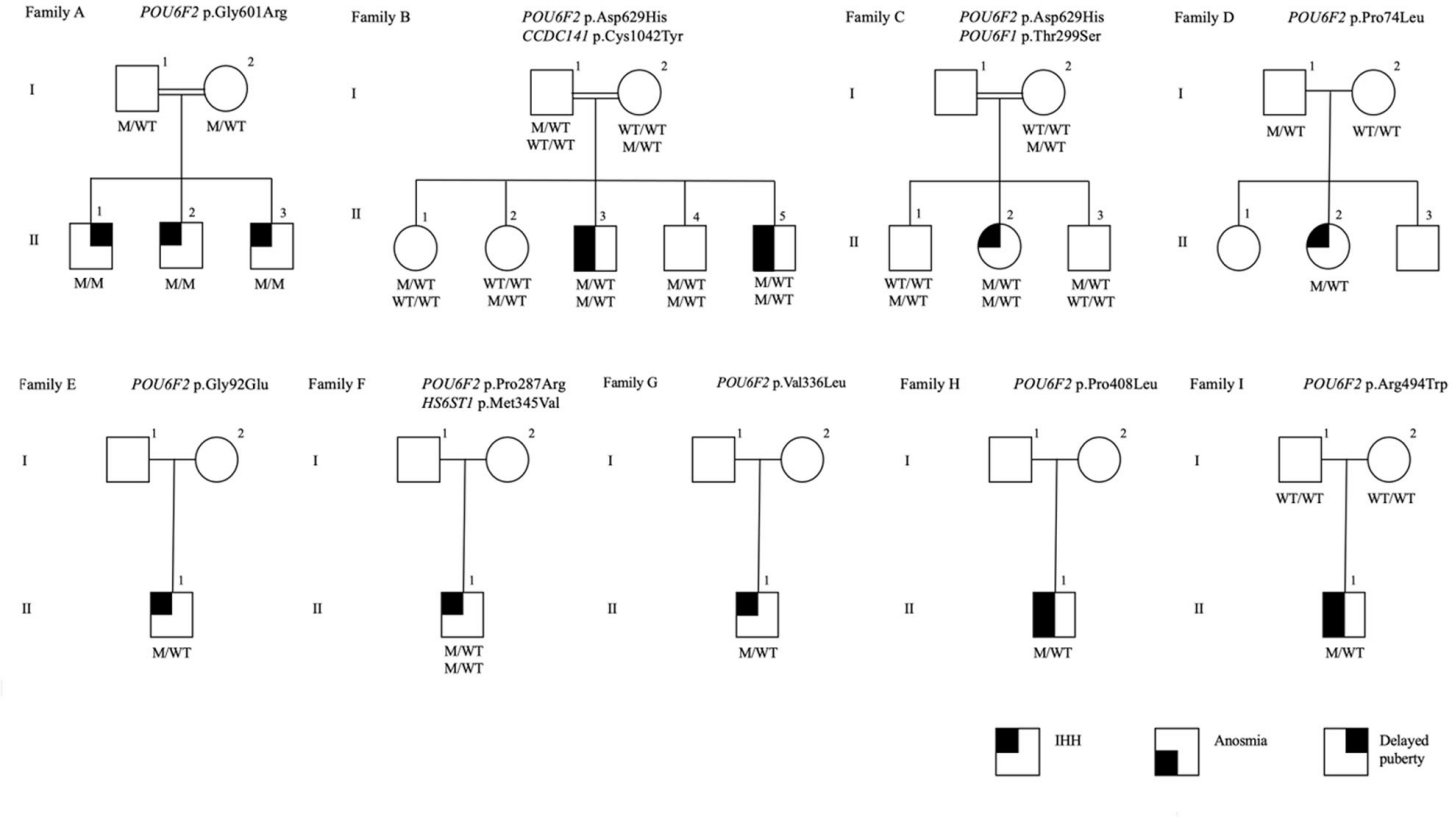
The pedigrees of the families with POU6F2 variants. Affected male and female family members are represented by black squares and black circles, respectively. White square symbols indicate unaffected male family members, white circle symbols represent unaffected female family members, and the double line indicates consanguinity. Under each symbol are the genotypes in the same order as the gene and variant descriptions, with WT and M denoting wild type and mutant, respectively. The legend denotes phenotypes as IHH, anosmia, and delayed puberty.

**Figure 2:**
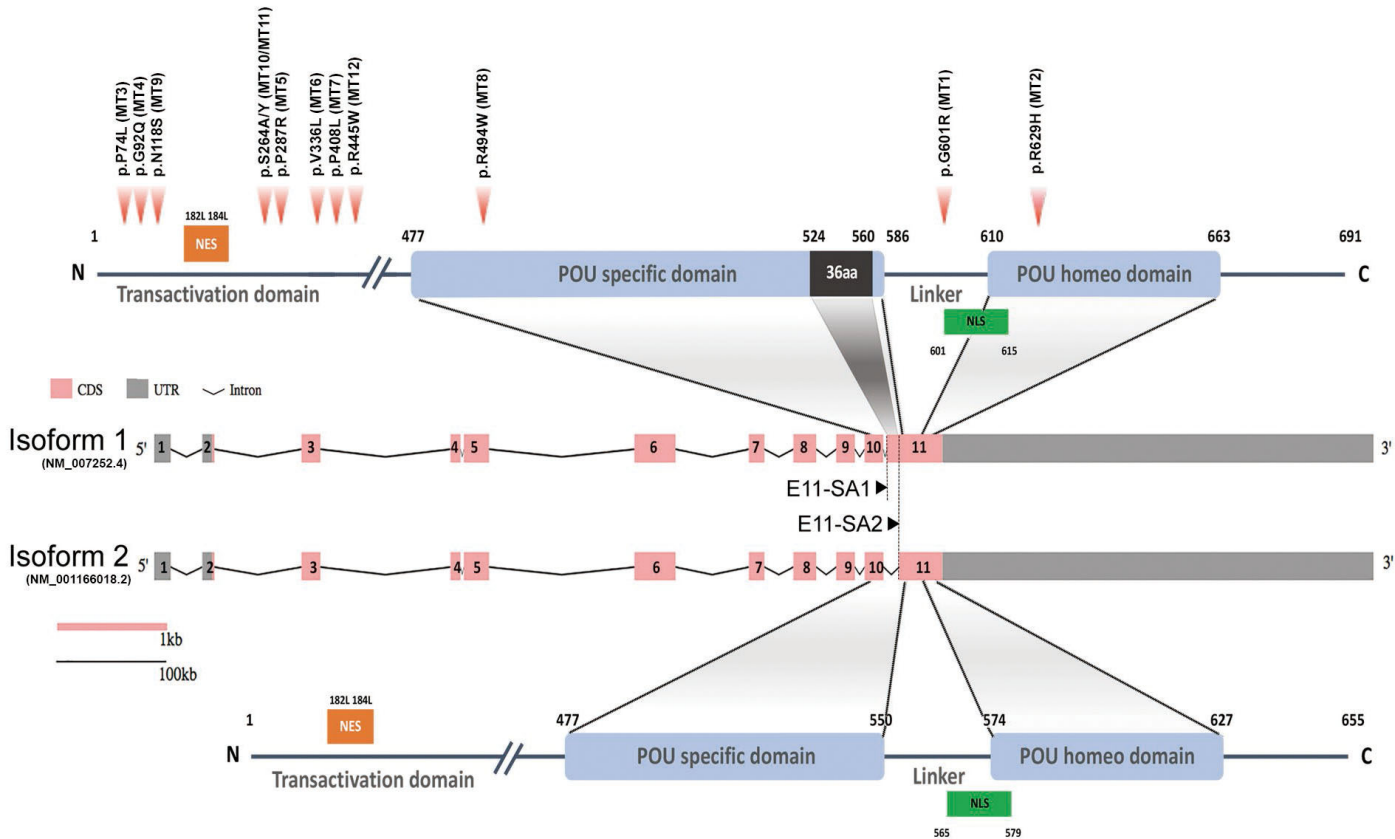
Schematic diagram of human *POU6F2* isoforms. Exon-intron structure of human *POU6F2* isoforms (middle two schematics) drawn to scale using the Gene Structure Display Server (GSDS 2.0, http://gsds.gao-lab.org). Exons are indicated by boxes to highlight the coding sequence (CDS, pink) and untranslated region (UTR, gray). Introns are indicated by black lines with a shrinked scale (0.01 ratio to scale of exons). Exon 11 is alternatively spliced *via* two splicing acceptor sites, E11-SA1 and E11-SA2, to generate isoform1 (upper schematic) and isoform2 (lower schematic), respectively. The two conserved DNA-binding domains are indicated by blue boxes and aligned to exons (encoded by exon 10 to 11). Isoform1 has a unique 36aa insertion on POU-specific domain (black box) not found in any other POU protein family members. The amino acid numbers are shown at the start and end point of functional domains. Twelve variants identified from IHH patients are indicated by red arrowheads (upper schematic). Mutation 1 (MT1; c.1801G>A, p.G601R in isoform1; c.1693G>A, p.G565R in isoform2) is in the linker region between the two DNA-binding domains. MT2 (c.1885A>C, p.N629H in isoform1; c.1777A>C, p.N593H in isoform2) is in the POU homeodomain. MT3–MT7, MT9–MT12 are in the Transactivation domain. MT8 (c.1480C>T, p.R494W) is in the POU-specific domain. Orange boxes; Nuclear export signal (NES), green boxes; Nuclear localization signal (NLS).

Twelve rare missense *POU6F2 variants* (HGNC: 21694) in 15 patients from 12 unrelated families were identified. The pedigrees with clinical phenotypical features are depicted in **Figure 1** and **Table 1**. Molecular genetic characteristics of the variants are shown in **Table 2**. Three POU domain variants (MT1, MT2, MT8) reside in regions necessary for proper protein function or dimerization (**Figure 2**). The remaining variants (MT3–MT7) are in the transactivation domain. Ten of the 12 variants had CADD scores >20 and either were not seen in the largest reference population database (gnomAD) or occurred at an extremely rare minor allele frequency <0.0005. However, MT2 was found to be significantly more common in the newly published Turkish Variome at 0.002 (14) (**Table 2**). No variant was previously reported in ClinVar. All were classified as variants of uncertain significance (VUS), except MT4 and MT7 that were categorized as “likely pathogenic” by ACMG/AMP classification (30). However, Polyphen-2 (31) and SIFT (32), two well-validated *in silico* prediction programs, indicated most of these variants to be harmful (**Table 2**). No other potentially harmful variants in the known IHH-associated genes other than those listed in **Figure 1** and **Table 1** were found.

**Table 1:** Clinical characteristics of individuals with POU6F2 variants.

Family/individual no.	Variant	Age at diagnosis (years)	Sex	Ethnicity	Initial basal LH (mIU/ml) /Estradiol (ng/dl) or Testosterone (ng/dl)	Stimulated maximum LH (mIU/ml)	Olfaction	Reproductive phenotype
A II-1	p.Gly601Arg	15	M	Turkish	NA	NA	Normosmic	Delayed puberty, Constitutional delay in growth and puberty
A II-2	p.Gly601Arg	18	M	Turkish	NA	NA	Normosmic	Absent puberty, Infertility
A II-3	p.Gly601Arg	14	M	Turkish	0.14/<10	0.98	Normosmic	Absent puberty
B II-3	p.Asn629His	15	M	Turkish	<0.1/<10	3.3	Anosmic	Absent puberty, cryptorchidism
B II-5	p.Asn629His	0.1	M	Turkish	<0.1/<10	0.78	NA	Absent mini puberty, microphallus 1 cm, cryptorchidism
C II-2	p.Asn629His	17	F	Arabic	0.2/0.4	0.2	Normosmic	Absent puberty, primary amenorrhea
D II-2	p.Pro74Leu	16	F	Turkish	0.1/1.1	0.1	Normosmic	Absent puberty, primary amenorrhea
E II-1	p.Gly92Glu	16	M	Turkish	0.1/NA	<10	Normosmic	Absent puberty
F II-1	*p.Pro287Arg*	17	M	Turkish	<0.1/<12	3.6	Normosmic	Absent puberty
G II-1	p.Val336Leu	18	M	American Caucasian	NA	NA	Normosmic	Absent puberty
H II-1	p.Pro408Leu	18	M	American Caucasian	NA	NA	Anosmic	Absent puberty, cryptorchidism
I II-1	p.Arg494Trp	18	M	Ashkenazi Jew	NA	NA	Anosmic	Absent puberty
J II-1	p.Asn118Ser	20	M	Turkish	NA	NA	Normosmic	Absent puberty
K II-1	p.Ser264Ala p.Ser264Tyr	35	M	Ashkenazi Jew	NA	NA	Normosmic	Absent puberty
L II-1	p.Arg445Trp	18	M	Asian	NA	NA	Anosmic	Absent puberty, microphallus at birth, cryptorchidism

**Table 2:** The molecular genetic characteristics of the *POU6F2* variants.

Family /individual no.	Variant name	Variant at cDNA level	Variant at protein level	TRV AC	TRV MAF	GnomAD MAF	CADD score	GERP	PP2	SIFT	ACMG/AMP	Other IHH gene variant /zygosity
A II-1, II-2, II-3	MT1	c.1801G>A	p.Gly601Arg	1 het /5170	0.000177	0.000017	28.8	5.28	D	D	VUS: PM1, PP2, PP3	None
B II-3, II-5	MT2	c.1885A>C	p.Asn629His	16 hets /6724	0.0023	0.000439	23.6	2.81	D	D	VUS: PM1, PP2, PP3	*CCDC141* p.Cys1042Tyr Het
C II-2	MT2	c.1885A>C	p.Asn629His	16 hets /6724	0.0023	0.000439	23.6	2.81	D	D	VUS: PM1, PP2, PP3	*POU6F1* p.Thr299Ser Het
D II-2	MT3	c.221C>T	p.Pro74Leu	0	0	0.000021	25.1	5.84	D	D	VUS: PP2	None
E II-1	MT4	c.275G>A	p.Gly92Glu	0	0	0.000007	27.8	5.84	D	D	LP: PM2, PP2, PP3	None
F II-1	MT5	c.860C>G	p.Pro287Arg	0	0	–	25.7	4.24	D	D	VUS: PM2, PP2	*HS6ST1* p.Met345Val Het
G II-1	MT6	c.1006G>C	p.Val336Leu	1 het /5174	0.000177	0.000027	23.8	6.17	D	T	VUS: PM1, PP2	None
H II-1	MT7	c.1223C>T	p.Pro408Leu	0	0	0.000011	31.0	5.62	D	D	LP: PM1, PM2, PP2, PP3	None
I II-1	MT8	c.1480C>T^*^	p.Arg494Trp	0	0	0.000020	34.0	5.48	D	D	VUS: PM1, PP2, PP3	None
J II-1	MT9	c.353A>G	p.Asn118Ser	0	0	–	19.9	5.02	T	D	VUS: PM1, PM2, PP2	None
K II-1	MT10	c.790T>G	p.Ser264Ala	0	0	0.000024	16.2	2.31	T	D	VUS: PM1, PM2, PP2	None
	MT11	c.791C>A	p.Ser264Tyr	0	0	0.000024	23.9	4.66	D	D	VUS: PM1, PM2, PP2	
L II-1	MT12	c.1333C>T	p.Arg445Trp	0	0	0.000032	26.7	5.70	D	D	VUS: PM1, PP2	None

Pro-to-Leu change at 74 (MT3) or 408 (MT7) could shift the hydrophilic/hydrophobic balance of this section of the protein toward hydrophobicity. Gly92 (MT4) is conserved in all the orthologs and most paralogs. This Gly-to-Glu variant, which is predicted likely pathogenic, could add a strong ionic charge that is normally absent in its vicinity. Pro287(MT5) is embedded in a short proline-rich region, which is well conserved. Mutations in this region have been implicated in prolactinoma (p.Pro280Leu and Gly292Ser) and Wilms tumor susceptibility (p.Ser270Pro and p.Pro273Leu) (Miao et al., 2019). Val336 (MT6) is well conserved in orthologs and partly conserved in paralogs. This Val-to-Leu variant is an amino acid substitution in the same hydrophobic group, so it is predicted as a tolerant variation from the SIFT but still deleterious from PP2 prediction.

Het, heterozygous; AC, allele count; MAF, minor allele frequency; TRV, Turkish Variome; GnomAD, The Genome Aggregation Consortium; InterVar, Interpretation of genetic variants by the ACMG/AMP 2015; VUS, variant uncertain significance; LP, likely pathogenic; PM, pathogenic moderate; PP, pathogenic supporting; CADD, Combined Annotation Dependent Depletion; GERP, Genomic Evolutionary Rate Profiling. Variants are described according to the RefSeq numbers following the gene names: POU6F2, NM_007252; CCDC141, NM_173648; POU6F1, NM_001330422; HS6ST1, NM_004807. PolyPhen-2, Polymorphism Phenotyping v2; SIFT, Sorting Intolerant From Tolerant; D, deleterious; T, tolerated.

*A *de novo* variant.

In Family-A, three brothers born from a consanguineous union presented with pubertal failure implicating an autosomal recessive mode of inheritance. All three brothers carried a homozygous variant (p.Gly601Arg). The two younger siblings had complete IHH. The oldest sibling received monthly testosterone injections at 15 years because of pubertal delay and by age 17 had started puberty. On a physical examination at age 24, he had testicular volumes of 25 ml bilaterally. Different from his brothers, the milder reproductive phenotype of this patient is consistent with constitutional delay in growth and puberty, also known as self-limited delayed puberty. It has been previously observed that variants in IHH genes can also cause self-limited delayed puberty, even sometimes within the same kindreds, indicating that self-limited delayed puberty shares an underlying pathophysiology with IHH (33, 34). The pattern of inheritance in Family-A is clearly autosomal recessive (**Figure 1**). MT8 (p.Arg494Trp) in Family-I arose *de novo*. A perfect segregation of an autosomal recessively inherited variant with pubertal failure phenotype in multiplex families such as in Family-A was given high scores in the Clinical Genome Resource (ClinGen) framework to define and evaluate the validity of gene–disease pairs across a variety of Mendelian disorders (35). Likewise, the *de novo* variant in Family-I provides strong genetic evidence supporting causality of mutations in novel gene–disease associations (35).

The inheritance in the other pedigrees is consistent with autosomal dominant with variable penetrance and expressivity, a phenomenon commonly observed in IHH (2, 36, 37). The male patients in Family-B, Family-H, and Family-L had cryptorchidism, indicating severe congenital hypogonadism. In congenital IHH, fetal pituitary gonadotropin secretion is low, leading to inadequate fetal serum testosterone levels. As the testicular descent and growth of phallus are androgen-dependent during fetal and neonatal periods, boys with severe IHH present with micropenis and/or cryptorchidism at birth (38). The younger patient in Family-B was diagnosed with IHH based on hypogonadal features plus prepubertal gonadotropins and testosterone level at 2 months of age, a time window known as minipuberty, a poorly understood transient activation of the hypothalamic-pituitary-gonadal (HPG) axis between 2 and 6 months of age. With an appropriate physical examination and laboratory findings, it is possible to make a diagnosis of IHH during this very early window of human life (39).

In Family-C, the 17-year-old female proband has the same variant as the one in Family-B. In addition, she carries a distinct rare variant in another POU family gene, *POU6F1* (see *Discussion* for a detailed assessment). The probands in the remaining eight families (other than in families A, B, C, and H) had variants in the non-POU domain part of the gene (**Figure 2**), the function of which remains poorly defined. We did not perform functional studies on these non-POU domain variants. However, these extremely rare variants were predicted to be deleterious by *in silico* analysis (**Table 2**).

Three variants (MT1, MT2, and MT8) are in the POU-specific domain (POU_S_, MT8), the linker region between the POU_S_ and the POU homeodomain (POU_H_, MT1), or in the POU_H_ (MT2, **Figure 2A**) (8, 40). R494 (MT8) is in the first alpha helix of the POU_S_ domain that is highly conserved in orthologs and conserved among paralogs as positively charged amino acids R or K. As such, a mutation changing R to W may alter the structure of this alpha helix. However, data from other POU family members indicate that it is residues of the third alpha helix in the POU_S_ domain that are involved in hydrogen bonding with DNA base pairs (41). As such, we performed *in silico* analysis (**Supplementary Figure S1**) but not functional studies of MT8. In contrast to MT8, R601 (MT1) and N629 (MT2) are located at/or close to the edge of alpha helixes that compose the POU_H_ domain, and these are less conserved among paralogs but well conserved in orthologs. Notably, MT1 and MT2 were the most prevalent variants identified, found in five of the 15 patients with POU6f2 variants and are on exon 11, which is alternatively spliced to form isoform1 and isoform2 (**Figure 2**). As such, functional studies were performed on MT1 and MT2 (see below).

### Pou6f2 isoforms are differentially expressed in mouse hypothalamic-pituitary-gonadal axis tissue

**Figure 3:**
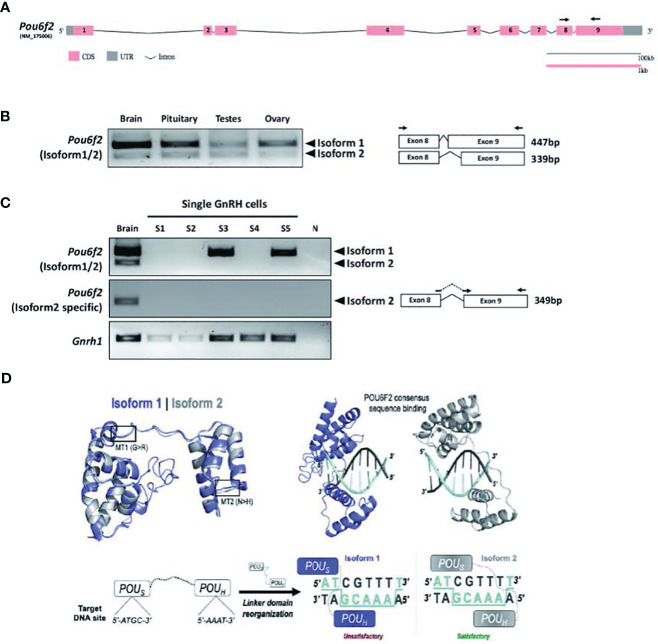
Expression of *Pou6f2* isoforms in mouse and bioinformatic prediction of POU6F2 isoforms bound to a DNA octamer. **(A)** Exon-intron structure of mouse *Pou6f2* (GSDS 2.0, http://gsds.gao-lab.org). In mice, only one isoform has been reported that is composed of nine exons and corresponds to isoform1 of human *POU6F2*. Primers used for PCR are shown as arrows on exon 8 and 9. **(B)** Gel image of RT-PCR analysis performed in mouse tissue. Top band (447 bp) shows isoform1 and bottom band (339bp) shows isoform2, which is skipping 108 bp by alternative splicing on exon 9. **(C)** Gel image of RT-PCR analysis of *Pou6f2* isoforms (top and middle gel) in GnRH single cells (bottom gel). A robust signal for GnRH was detected in three cells, and in two of these cells, only isoform1 was detected. Isoform2 was not detected in any of the GnRH cells. **(D)** Upper Left, Superimposition of isoform1 (purple) and isoform2 (gray) structures predicted by C-I-TASSER. The location of MT1 and MT2 is indicated by boxes. Upper Right, HDOCK prediction of POU6F2 binding to the OCT1 DNA consensus site (5’-ATGCAAAT-3’). Template-free docking was used to prevent simulation bias. Lower Left and Right, Structural representation of the interaction between each isoform and dsDNA octamers. Two-dimensional cartoon illustrating the molecular interactions between each POU domain and their predicted binding sites. Satisfactory (for isoform2) and unsatisfactory (for isoform1) binding modes are indicated.

To determine which isoform might be pertinent to patients exhibiting IHH and thus our functional studies on the variants, the expression of POU6F2 isoforms in HPG axis-relevant mouse tissues was performed using RT-PCR (**Figure 3**). *Pou6f2* is well conserved between human and mouse, except with respect to the 5’ UTR, which is located on exon 1 and exon 2 in human, while mouse *Pou6f2* has a shorter 5’ UTR with its coding region starting from exon 1 (compare **Figure 2**, human and **Figure 3A**, mouse). Thus, mouse *Pou6f2* has nine exons that correspond to exon 3–11 in the human. To date, only one *Pou6f2* mRNA sequence has been cataloged in NCBI; however, the alternative splicing of the last exon was analyzed in mouse retina cDNA and revealed the presence of both isoform1 and isoform2 (40). In brain, pituitary, and gonads from mice (**Figure 3B**), both isoforms were present, although the expression of isoform1 was more abundant than that of isoform2. Analysis of primary mouse GnRH cells (**Figure 3C**) and two mouse GnRH cell lines (**Supplementary Figure S2**) with primers that detect both isoform1 and isoform2 showed isoform1 transcript present in cells that showed robust GnRH product. To ensure that isoform2 was not being missed due to low expression, the primary GnRH cells were rescreened with isoform2 only specific primers (**Figure 3C**). After 45 cycles of amplification, no isoform2 transcripts were detected in any of the GnRH cells, although brain cDNA was positive. Thus, in primary mouse GnRH cells, isoform1 is the predominant *Pou6f2* isoform that is expressed.

### POU6F2 modeling

Although POU6F2 has yet to be crystallized, a closely related paralog human POU6F1 has been crystallized bound to an octamer motif (41), which increases the accuracy of homology modeling (42). C-I-TASSER produced structures for POU6F2 isoform1 and isoform2 (**Figure 3D**) using the POU6F1 crystal template with good resolution (PDB code: 3D1N; resolution = 2.51 Å). The POU domains for each POU6F2 isoform were in the same fold as POU6F1 (TM-score_iso1_ = 0.66, RMSD_iso1_ = 1.03; TM-score_iso2_ = 0.80, RMSD_iso2_ = 0.81). Highly variant N-terminal domains upstream of the POU domains were not detected in the original POU6F1 crystal and were disordered in POU6F2 structures and thus were omitted in downstream structural experiments. *In silico* analysis was next used to validate our modeling. Previous experiments indicated that only isoform2 binds to OCT1 consensus DNA (8). w3DNA was used to predict the structure of the human OCT1 DNA consensus sequence (5’-A_1_T_2_G_3_C_4_A_5_A_6_A_7_T_8_-3’), and HDOCK predicted a more favorable scoring function of binding for isoform2 (-303.26au) compared to isoform1 (-258.88au). The predicted binding mode showed that isoform2 POU_S_ binds to 5’-A_1_T_2_G_3_C_4_-3’ and POU_H_ to 5’-A_5_A_6_A_7_T_8_-3’ by embracing both faces of dsDNA, whereas isoform1 did not (**Figure 3D**). This is consistent with literature (8), validating the structure of our models.

### Functional analysis of POU6F2 isoform1 as a potential transcription factor

**Figure 4:**
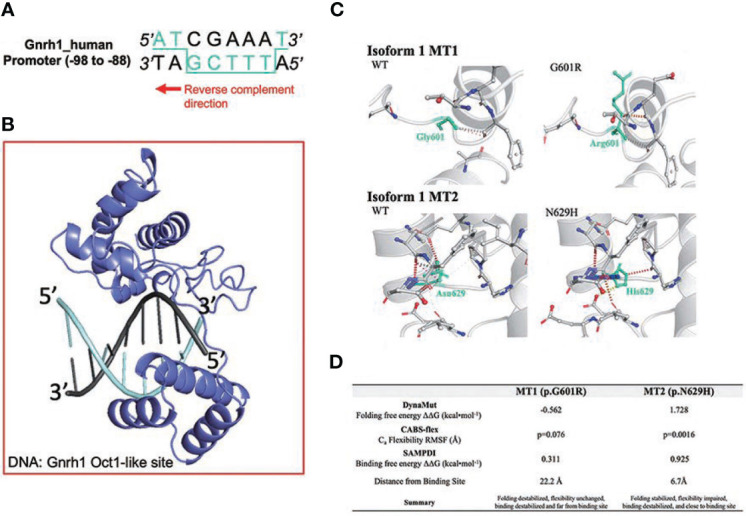
Structural analysis of IHH variants MT1 and MT2 on POU6F2 isoform1. **(A)** OCT1 consensus-like site (5’-ATGCTTTT-3’) is identified in human *GnRH1* promoter (-98 to -88). Binding site in 3D modeling uses POU_S_ to ATGC, and the POU_H_ is predicted to insert into a groove between both faces of the dsDNA, thus contacting both TTTT and AAAA. **(B)** HDOCK prediction of POU6F2 isoform1 binding to the OCT1 consensus-like site. Template-free docking was used to prevent simulation bias. **(C)** DynaMut prediction of WT and mutant proteins for isoform1. Individual amino acid substitutions are indicated in cyan. **(D)** Structural evaluation scores indicating how MT1 and MT2 affect POU6F2 isoform1 protein folding (DynaMut), natural protein flexibility (CABS-flex), and DNA binding (SAMPDI). DynaMut and CABS-flex represent changes in the individual protein structures, whereas SAMPDI represents changes in the affinity of POU6F2 isoform1 to bind the OCT1 consensus-like site (5’-ATGCTTTT-3’). Characterization of stabilizing or destabilizing effects are indicated. CABS-flex values analyzed using a paired t-test.

Compared to POU6F2 isoform2, the function of POU6F2 isoform1 is unclear. However, using the Yeast One-Hybrid System with a 5’-upstream region of the porcine *Fshβ* as the bait sequence, Yoshida et al. (10) cloned a cDNA encoding a partial sequence of the POU domain from porcine pituitary. The clone was equivalent to POU6F2 isoform1 and was able to modulate the expression of developmental pituitary genes using transient transfection assays of promoter activity in CHO cells. We tested our modeled isoform1 against the predicted *Fshβ* protected site (5’-ATAAGCTTAAT-3’) and found that not only does isoform1 bind in the correct orientation (i.e., insert facing away from DNA) but the POU_S_ binds onto ATAA and POU_H_ onto TTAA, which agrees with the sites that POU6F1 monomer2 uses to bind CRH (crystal PDB code: 3D1N). Examining the *GnRH1* promoter, we found a similar site but with three mismatches (AAAAGCATAGT, region of *GnRH1* promoter sequence that aligned with *Fshβ*). When we tested this site using HDOCK, isoform1 did not interact with the correct domains/orientation and the docking solutions were not in agreement with the binding mode predicted with *Fshβ.* However, we noticed that the *GnRH1* promoter region contains a reverse-complementary version of the POU6F2 consensus site with one A/T substitution (POU6F2 consensus: 5’-ATGCAAAT-3’; *GnRH1* site: 5’-TACGAAAA-3’ = 3-ATGCTTTT-5’, **Figure 4A**). Using 3D modeling, one sees the POU6F2 consensus site arrangement and appropriate binding for isoform1 (**Figure 4B**, i.e., POUs to ATGC half and POU_H_ to AAAA half), which is in fact in agreement with isoform2. The 44-nucleotide insert on isoform1 sticks away from the complex allowing it to bind. Next, DynaMut and CABS-flex were used to determine changes in protein structure that might be induced by MT1 and MT2 on isoform1 (**Figures 4C, D**). DynaMut predicted that MT1 destabilized while MT2 stabilized isoform1 folding (ΔΔ_GMT1_ = -0.562; ΔΔG_MT2 _= 1.728). CABS-flex revealed that isoform1 protein flexibility was decreased only with MT2 (P = 0.0016).

**Figure 5:**
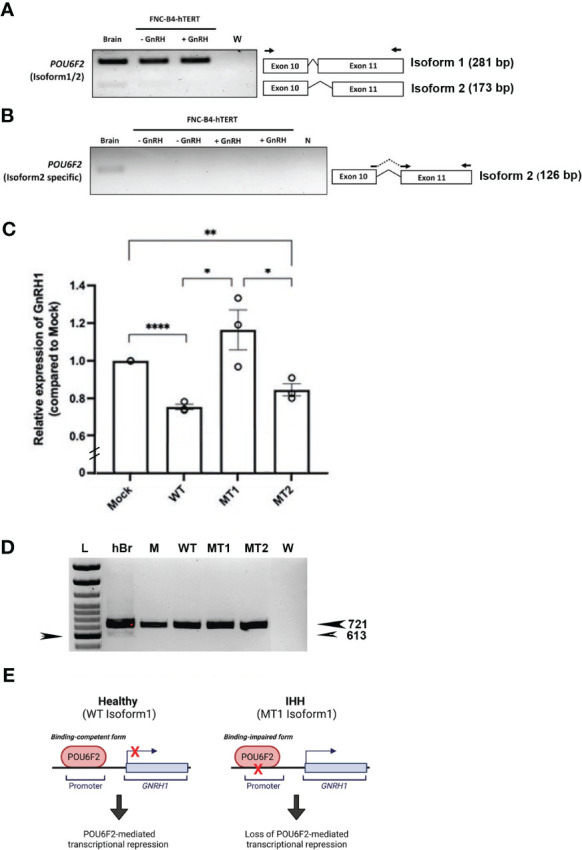
*In vitro* transcription assay of isoform1 in immortalized human GnRH cells. **(A)** Expression of POU6F2 isoforms in human brain and FNC-B4-hTERT cells. RT-PCR analysis performed in human brain and immortalized human GnRH cells (with or without GnRH stimulation). Top band (281 bp) shows isoform1 in all tissue samples. In human brain, a bottom band (173 bp) is detected, isoform2 that is skipping 108 bp by alternative splicing on exon 11. Primers used for PCR are shown as arrows on exon 10 and 11. **(B)** Nested RT-PCR analysis performed using isoform2-specific primers (shown as arrows on the junction of exon 10–11 and exon 11). Consistent with the first run, isoform2 (126 bp) was only detected in human brain not in the FNC-B4-hTERT cells. **(C)** Quantitative RT-PCR of *GnRH1* in FNC-B4-hTERT cells transfected with POU6F2 isoform1s (WT, MT1, MT2). The expression of *GnRH1* was normalized to each experimental Mock group (plasmid only, relative expression level = 1), and the relative values of the other three groups are shown in the bar graph. MT1 significantly increased *GnRH1* transcript compared to both WT and MT2 groups but was not significantly different from the Mock group. **(D)** RT-PCR for POU6F2 isoform2 in FNC-B4-hTERT cells transfected with POU6F2 isoform1 WT, MT1, and MT2. Consistent with non-transfected FNC-B4-hTERT cells **(A)**, each of the experimental groups expressed only isoform1. Since endogenous POU6F2 was still present in the transfected cells, our results suggest that overexpression of MT11 had a dominant-negative effect. cDNA in other lanes: hBr = human brain, M = mock, W = water. Arrow on left pointing to 600-bp band on ladder. **(E)** Schematic summary of isoform1 as a transcriptional regulator generated by Biorender (https://biorender.com/).

To directly evaluate the transcriptional activity of WT and mutant isoform1 POU6F2 proteins, *in vitro* transcription assays were performed using a human GnRH cell line FNC-B4-hTERT (21). Consistent with the results obtained in primary GnRH cells from mice (**Figure 3**) and two mouse GnRH cell lines (**Supplementary Figure S2**), isoform1 was robustly expressed in FNC-B4-hTERT cells and not isoform2 (**Figures 5A, B**). For the *in vitro* transcription assays, GnRH transcript was measured using qPCR (**Figure 5C**). The Mock ΔΔCт was set to 1 and the remaining treatment conditions were adjusted accordingly to compare across experimental runs. After transfection, the expression of either WT-POU6F2 or MT2-POU6F2 isoform1 in these cells significantly decreases GnRH expression compared to mock (Mock = 1; WT = 0.7547 ± 0.014, ****P < 0.0001; MT2 = 0.8458 ± 0.032, **P < 0.001). No significant difference was found between WT and MT2 isoform1-treated groups. Notably, MT1 significantly increased GnRH transcript compared to both WT and MT2 groups (MT1 = 1.164 ± 0.11, P < 0.05 for both comparisons) but was not significantly different from the Mock group. Since endogenous POU6F2 was still present in the transfected cells, our results suggest that overexpression of MT1 had a dominant-negative effect.

Since MT1 and MT2 reside in exon 11, known for containing splicing events that distinguish isoform1 and isoform2 (21, 40), the cDNAs from the same experimental groups assayed by qPCR were examined for POU6F2 isoform1 and isoform2 expression using standard RT-PCR. Consistent with nontransfected FNC-B4-hTERT cells (**Figure 5A**), each of the experimental groups expressed only isoform1 (**Figure 5D**). These experiments reveal that isoform1 POU6F2 proteins transcriptionally regulate *GnRH1* (**Figure 5E**) and that a variant identified in three patients with IHH (all normosmic) prevented the transcriptional regulation of *GnRH1*.

## Discussion

Our findings reveal (a) that GnRH cells express POU6F2 isoform1, (b) that this isoform can act as a repressor (negative transcription factor) for *GnRH1*, and (c) that a variant of POU6F2 identified in three IHH patients (MT1) blocks the repression of *GnRH1* expression. The action of POU6F2 isoform1 acting as a transcription factor is a novel finding and supports the clinical and molecular genetics data that highlighted POU6F2 variants in 15 patients from 12 independent families who all presented with pubertal failure and were diagnosed with IHH. The lack of an effect by MT2 is consistent with new Turkish variome data (14) (unlike that in GnomAD), indicating that this variant is probably too common in the Turkish population to cause IHH by itself but may contribute to the phenotype in combination with other variants. Notably, patients in Family-B and Family-C carry MT2, but both had additional variants in other genes. Patients in Family-B possessed a rare heterozygous variant in *CCDC141*, which encodes for a protein involved in embryonic GnRH neuron migration (43) and is a known IHH-causative gene (44). Thus, the co-occurrence of the rare variant in *CCDC141* may explain IHH in these kindred. The proband in Family-C had rare heterozygous variants in POU6F1. Although little is known about the significance of the site of the POU6F1 variants, it is possible that the combination of these variants in the closest paralogs to POU6F2 had an integrated effect to cause the IHH phenotype in this patient.

Since IHH patients have low gonadotropins in the face of prepubertal serum sex steroid levels, the pathophysiology of this condition should reside in the pituitary and/or hypothalamus. POU6F2 has been shown to be highly expressed in the early embryonic pituitary and stimulate the expression of PROP1 (10), although the specific isoforms were not examined. PROP1 is well known to induce POU1F1 and the development of gonadotropes and corticotropes in the anterior pituitary (45). POU1F1 also induces differentiation of GH-, PRL-, and TSHB-producing cell lineages in the anterior pituitary (3) and, when mutated, causes multiple pituitary hormone deficiency syndrome and hypopituitarism (46). However, it is unlikely that IHH in our patients is due to impaired pituitary effects of POU6F2 *via* PROP1, since our patients have only a deficiency of LH and FSH and not ACTH or any of the remaining three pituitary hormones (growth hormone, prolactin, and TSH) induced by POU1F1 after PROP1 stimulation.

There are many modulators of reproduction within the hypothalamus, but most are translated to the pituitary–gonadal axis *via* GnRH neurons, and dysregulation of GnRH neurons prenatally or postnatally can result in an altered HPG axis. In this report, we show that WT-POU6F2-isoform1 can directly inhibit *GnRH1* transcription and that MT1 alters the transcriptional activity of this isoform. The recent crystallization of POU6F1 revealed that members of the POU6 family can bind target DNA as dimers such that the POU_S_ and POU_H_ domains of one monomer bind opposite faces of dsDNA *via* a flexible linker region and that the POU_S_ domain of the other monomer binds adjacent to the first POU_S_ (41). POU6F2 isoform1 was shown to interact with a region of the *FSHβ* promoter (10). Although a similar region (three nucleotide changes) was found in the GnRH promoter, modeling did not show binding. However, an OCT1 consensus-like site (5’-ATGCTTTT-3’) was identified in the human *GnRH1* promoter (-99 to -92). Three-dimensional modeling predicted that the POU_S_ bound to ATGC, and the POU_H_ inserted into a groove between both faces of the dsDNA, contacting both the TTTT and AAAA. Thus, the results of computational modeling and quantitative RT-PCR of *hGnRH1* are consistent with POU6F2 isoform1 binding to the *GnRH1* promoter and acting as a negative regulator.

Prenatally, GnRH cells migrate from the olfactory placode into the developing forebrain. Alterations in GnRH expression occur during migration with the cells pausing at the nasal forebrain junction (47). As they enter the forebrain, there is a significant increase in GnRH transcription (48) with concomitant changes in protein expression (16, 49) as well as neuronal activity (47). Previous studies in mouse showed that MSX and DLX, non-Hox homeodomain transcription factors, compete for the same binding site and alter GnRH transcription differently, with DLX enhancing and MSX repressing GnRH expression (50). The authors reported that MSX mutant mice had more GnRH-expressing cells at E13.5, and that most of these cells were confined to nasal regions being distributed in both expected regions as well as ectopically in the olfactory epithelium. In addition, the study reported that the mouse GN11 cell line, a model for immature migrating GnRH cells, expressed MSX, while the GT1-7 cells, a model for mature mouse GnRH cells, expressed both DLX and MSX. To date, one knockout *POU6F2* animal model has been reported that removed exon 11 and examined central corneal thickness where isoform2 is robustly expressed (9). No other phenotypes were described. To examine the role of isoform1 and some of the variants associated with IHH patients, we chose to use a human GnRH cell line that is derived from olfactory mucosa, representing an immature GnRH cell. Overexpression of WT-POU6F2-isoform1 repressed GnRH expression in these cells, correlating with low GnRH transcription levels observed when GnRH cells are outside the forebrain, prioritizing migration over maturation. As such, mutations releasing POU6F2 isoform1 repression, like MT1, increase GnRH expression that, as observed in MSX mutants (50), likely results in early cell cessation. This event would be detrimental to the developing GnRH neuronal system and lead to IHH.

In the adult, POU6F2 is expressed in the dorsal hypothalamus in a scattered fashion (8, 10), which may overlap with the dispersed location of GnRH neurons in the hypothalamus (51). Other POU domain genes [POU3F1 also known as OCT6 (6, 7) and POU2F1 (also known as OCT1 (5)] have been shown to repress (6) or enhance (5, 7) *GNRH1* expression. Wierman et al. (6) speculated that POU3F1 is able to turn off and on the transcriptional machinery in postnatal GnRH cells, influenced by the hormonal environment (such as sex steroids), when groups of GnRH cells were reported to be unable to express the mature gene product (52). Certainly, POU6F2 isoform1 could play a similar role in GnRH cells postnatally, since the transcript was expressed in both NLTs and GT1-7 cells, two immortalized GnRH mouse cell lines often used as models for immature (19) and mature GnRH cells (18). As an alternative/additional mechanism of disease *via* mechanisms post-GnRH cell migration into the forebrain, the effects of POU6F2 variants to impair pubertal development may occur indirectly to GnRH cells *via* the arcuate (infundibular) nucleus. The arcuate kisspeptin neurons have been proposed as the hypothalamic GnRH pulse generator driving fertility (53), and Nagae et al. (54) recently provided direct evidence that kisspeptin neurons maintain gonadotropin pulses and folliculogenesis. Campbell et al. profiled gene expression in the arcuate nucleus of the hypothalamus in adult mice and found that *Pou6f2* is highly expressed with a subgroup of *Pomc* neurons, a major anorectic gene, which may also give rise to kisspeptin neurons (55, 56). In addition, a single-cell transcriptome analysis of the hypothalamic arcuate nucleus in E15 mouse showed that *Pou6f2* was one of the transcription factors showing differential expression among subclusters (57). Whether these cells also express isoform1 and, if so, what role it plays in these GnRH-modulating neurons remain to be determined.

Several identified POU6F2 variants remain to be examined, with most occurring in the transactivation domain. These regions are known to act as transcription factor scaffold domains containing binding sites for other proteins such as transcription coregulators (3). The N-terminal sequences of some POU factors can, in fact, mediate repression, including regions from the N terminus of Oct-2 and Oct-3 (3). Of the eight *POU6F2* variants identified in the transactivating domain, three are associated with anosmia, suggesting a role in GnRH neuronal development either directly or indirectly *via* changes in the olfactory system. An appropriate model is needed to address the impact of these variants on normal POU6F2 function. The FNC-B4 cell line, post-immortalization, showed dose-dependent changes in migration to chemoattractants (21). Here, we show that this cell line expresses only POU6F2 isoform1 consistent with our finding in primary GnRH cells in mice. Thus, this cell line may provide a suitable model for future experiments to evaluate the functional significance of several of the variants identified in this paper occurring in the transactivating domain.

In summary, we provide evidence implicating variants in POU6F2 in the etiology of IHH with mutations in POU6F2 isoform1 directly impacting the GnRH expression.

## Data availability statement

The data presented in the study are deposited in the ClinVar repository, accession numbers VCV002499165.1, VCV002499168.1, VCV002499167.1, VCV002499166.1, VCV002499169.1.

## Ethics statement

Human experimental protocols were approved by either the Ethics Committee of the Cukurova University Faculty of Medicine and the institutional review board of the University of Mississippi Medical Center or by the Human Research Committee at the MGH, Boston, MA. All individuals and/or their legal guardians provided written informed consent to participate in this study. The animal study was reviewed and approved by NINDS Animal Care and Use Committee and performed in accordance with NIH guidelines.

## Author contributions

FG, MS, and LK participated in the genetic study. FG, MS, RB, SC, JM, SA, CG, GC, FB, IT and BY collected patients and clinical data. AT, FG, and LK designed and conducted genetic experiments, analyzed data, interpreted the results. SW, H-JC and SF designed and conducted cellular, bioinformatic, molecular and mutational functional experiments, analyzed data, interpreted the results. KB participated in *in vitro* splicing assay and transcription assay. MT participated in *in vitro* transcription assay. SW, H-JC, RB, SS, SF, LK and AT supervised the studies and wrote the manuscript. SW, RB, SS, and AT provided funding for these studies. All authors contributed to the article and approved the submitted version.

## Supplementary material

Supplementary Figure 1
*In silico analysis* of MT8.

Supplementary Figure 2 Gel image of RT-PCR analysis performed on two GnRH mouse cells lines (18, 19). Top band (447 bp) shows isoform1 and bottom band (339bp) shows isoform2 which is skipping 108bp by alternative splicing on exon 9. Both cell lines expressed only isoform1. 4div explant= nasal explant (16, 17) was used as a positive control.

## References

[1] Howard SR Dunkel L. Delayed puberty-phenotypic diversity, molecular genetic mechanisms, and recent discoveries. Endocr Rev (2019) 40(5):1285–317. doi: 10.1210/er.2018-00248 PMC6736054 31220230

[2] Louden ED Poch A Kim HG Ben-Mahmoud A Kim SH Layman LC. Genetics of hypogonadotropic hypogonadism-human and mouse genes, inheritance, oligogenicity, and genetic counseling. Mol Cell Endocrinol (2021) 534:111334. doi: 10.1016/j.mce.2021.111334 34062169

[3] Andersen B Rosenfeld MG. POU domain factors in the neuroendocrine system: lessons from developmental biology provide insights into human disease. Endocr Rev (2001) 22(1):2–35. doi: 10.1210/edrv.22.1.0421 11159814

[4] Kim KP Han DW Kim J Schöler HR. Biological importance of OCT transcription factors in reprogramming and development. Exp Mol Med (2021) 53(6):1018–28. doi: 10.1038/s12276-021-00637-4 PMC8257633 34117345

[5] Leclerc GM Boockfor FR. Identification of a novel OCT1 binding site that is necessary for the elaboration of pulses of rat GnRH promoter activity. Mol Cell Endocrinol (2005) 245(1-2):86–92. doi: 10.1016/j.mce.2005.10.026 16337733

[6] Wierman ME Xiong X Kepa JK Spaulding AJ Jacobsen BM Fang Z. Repression of gonadotropin-releasing hormone promoter activity by the POU homeodomain transcription factor SCIP/Oct-6/Tst-1: a regulatory mechanism of phenotype expression? Mol Cell Biol (1997) 17(3):1652–65. doi: 10.1128/mcb.17.3.1652 PMC231890 9032292

[7] Wolfe A Kim HH Tobet S Stafford DE Radovick S. Identification of a discrete promoter region of the human GnRH gene that is sufficient for directing neuron-specific expression: a role for POU homeodomain transcription factors. Mol Endocrinol (2002) 16(3):435–49. doi: 10.1210/mend.16.3.0780 11875100

[8] Zhou H Yoshioka T Nathans J. Retina-derived POU-domain factor-1: a complex POU-domain gene implicated in the development of retinal ganglion and amacrine cells. J Neurosci (1996) 16(7):2261–74. doi: 10.1523/JNEUROSCI.16-07-02261.1996 PMC6578531 8601806

[9] King R Struebing FL Li Y Wang J Koch AA Bailey JNC. Genomic locus modulating corneal thickness in the mouse identifies POU6F2 as a potential risk of developing glaucoma. PloS Genet (2018) 14(1):e1007145. doi: 10.1371/journal.pgen.1007145 29370175 PMC5784889

[10] Yoshida S Ueharu H Higuchi M Horiguchi K Nishimura N Shibuya S. Molecular cloning of rat and porcine retina-derived POU domain factor 1 (POU6F2) from a pituitary cDNA library. J Reprod Dev (2014) 60(4):288–94. doi: 10.1262/jrd.2014-023 PMC4139503 24804940

[11] DiRenzo F Doneda L Menegola E Sardella M De Vecchi G Collini P. The murine Pou6f2 gene is temporally and spatially regulated during kidney embryogenesis and its human homolog is over-expressed in a subset of wilms tumors. J Pediatr Hematol Oncol (2006) 28(12):791–7. doi: 10.1097/MPH.0b013e31802d3e65 17164647

[12] Miao Y Li C Guo J Wang H Gong L Xie W. Identification of a novel somatic mutation of POU6F2 by whole-genome sequencing in prolactinoma. Mol Genet Genomic Med (2019) 7(12):e1022. doi: 10.1002/mgg3.1022 31692290 PMC6900357

[13] Perotti D De Vecchi G Testi MA Lualdi E Modena P Mondini P. Germline mutations of the POU6F2 gene in Wilms tumors with loss of heterozygosity on chromosome 7p14^†^ . Hum Mutat (2004) 24(5):400–7. doi: 10.1002/humu.20096 15459955

[14] Kars ME Basak AN Onat OE Bilguvar K Choi J Itan Y. The genetic structure of the Turkish population reveals high levels of variation and admixture. Proc Natl Acad Sci USA (2021) 118(36):e2026076118. doi: 10.1073/pnas.2026076118 34426522 PMC8433500

[15] Topaloglu AK Turan I. Genetic etiology of idiopathic hypogonadotropic hypogonadism. Endocrine (2022) 3(1):1–15. doi: 10.3390/endocrines3010001

[16] Kramer PR Krishnamurthy R Mitchell PJ Wray S. Transcription factor activator protein-2 is required for continued luteinizing hormone-releasing hormone expression in the forebrain of developing mice. Endocrinology (2000) 141(5):1823–38. doi: 10.1210/endo.141.5.7452 10803593

[17] Constantin S. Physiology of the gonadotrophin-releasing hormone (GnRH) neurone: studies from embryonic GnRH neurones. J Neuroendocrinol (2011) 23(6):542–53. doi: 10.1111/j.1365-2826.2011.02130.x PMC3101116 21443528

[18] Mellon PL Windle JJ Goldsmith PC Padula CA Roberts JL Weiner RI. Immorta-lization of hypothalamic GnRH neurons by genetically targeted tumorigenesis. Neuron (1990) 5(1):1–10. doi: 10.1016/0896-6273(90)90028-e 2196069

[19] Radovick S Wray S Lee E Nicols DK Nakayama Y Weintraub B. Migratory arrest of gonadotropin-releasing hormone neurons in transgenic mice. PNAS. (1991) 88(8):3402–6. doi: 10.1073/pnas.88.8.3402 PMC51455 2014260

[20] Romanelli RG Barni T Maggi M Luconi M Failli P Pezzatini A. Expression and function of gonadotropin-releasing hormone (GnRH) receptor in human olfactory GnRH-secreting neurons: an autocrine GnRH loop underlies neuronal migration. J Biol Chem (2004) 279(1):117–26. doi: 10.1074/jbc.M307955200 14565958

[21] Hu Y Guimond SE Travers P Cadman S Hohenester E Turnbull JE. Novel mechanisms of fibroblast growth factor receptor 1 regulation by extracellular matrix protein anosmin-1. J Biol Chem (2009) 284(43):29905–20. doi: 10.1074/jbc.M109.049155 PMC2785620 19696444

[22] Zhang C Mortuz SM He B Wang Y Zhang Y. Template-based and free modeling of I-TASSER and QUARK pipelines using predicted contact maps in CASP12. Proteins (2018) 86 Suppl 1:136–51. doi: 10.1002/prot.25414 PMC5911180 29082551

[23] Zheng G Lu XJ Olson WK. Web 3DNA–a web server for the analysis, reconstruction, and visualization of three-dimensional nucleic-acid structures. Nucleic Acids Res (2009) 37(Web Server issue):W240–246. doi: 10.1093/nar/gkp358 PMC2703980 19474339

[24] Yan Y Tao H He J Huang SY. The HDOCK server for integrated protein-protein docking. Nat Protoc (2020) 15(5):1829–52. doi: 10.1038/s41596-020-0312-x 32269383

[25] Yan Y Zhan D Zhou P Li B Huang SY. HDOCK: a web server for protein-protein and protein-DNA/RNA docking based on a hybrid strategy. Nucleic Acids Res (2017) 45(W1):W365–73. doi: 10.1093/nar/gkx407 PMC5793843 28521030

[26] Rodrigues CH Pires DE Ascher DB. DynaMut: predicting the impact of mutations on protein conformation, flexibility and stability. Nucleic Acids Res (2018) 46(W1):W350–5. doi: 10.1093/nar/gky300 PMC6031064 29718330

[27] Kuriata A Gierut AM Oleniecki T Ciemny MP Kolinski A Kurcinski M. CABS-flex 2.0: a web server for fast simulations of flexibility of protein structures. Nucleic Acids Res (2018) 46(W1):W338–43. doi: 10.1093/nar/gky356 PMC6031000 29762700

[28] Peng Y Sun L Jia Z Li L Alexov E. Predicting protein-DNA binding free energy change upon missense mutations using modified MM/PBSA approach: SAMPDI webserver. Bioinformatics (2018) 34(5):779–86. doi: 10.1093/bioinformatics/btx698 PMC6048991 29091991

[29] Taylor S Wakem M Dijkman G Alsarraj M Nguyen M. A practical approach to RT-qPCR-Publishing data that conform to the MIQE guidelines. Methods (2010) 50(4):S1–5. doi: 10.1016/j.ymeth.2010.01.005 20215014

[30] Richards S Aziz N Bale S Bick D Das S Gastier-Foster J. Standards and guidelines for the interpretation of sequence variants: a joint consensus recommendation of the American college of medical genetics and genomics and the association for molecular pathology. Genet Med (2015) 17(5):405–24. doi: 10.1038/gim.2015.30 PMC4544753 25741868

[31] Adzhubei IA Schmidt S Peshkin L Ramensky VE Gerasimova A Bork P. A method and server for predicting damaging missense mutations. Nat Methods (2010) 7(4):248–9. doi: 10.1038/nmeth0410-248 PMC2855889 20354512

[32] Kumar P Henikoff S Ng PC. Predicting the effects of coding non-synonymous variants on protein function using the SIFT algorithm. Nat Protoc (2009) 4(7):1073–81. doi: 10.1038/nprot.2009.86 19561590

[33] Saengkaew T Ruiz-Babot G David A Mancini A Mariniello K Cabrera CP. Whole exome sequencing identifies deleterious rare variants in CCDC141 in familial self-limited delayed puberty. NPJ Genom Med (2021) 6(1):107. doi: 10.1038/s41525-021-00274-w 34930920 PMC8688425

[34] Zhu J Choa RE Guo MH Plummer L Buck C Palmert MR. A shared genetic basis for self-limited delayed puberty and idiopathic hypogonadotropic hypogonadism. J Clin Endocrinol Metab (2015) 100(4):E646–654. doi: 10.1210/jc.2015-1080 PMC4399304 25636053

[35] Strande NT Riggs ER Buchanan AH Ceyhan-Birsoy O DiStefano M Dwight SS. Evaluating the clinical validity of gene-disease associations: an evidence-based framework developed by the clinical genome resource. Am J Hum Genet (2017) 100(6):895–906. doi: 10.1016/j.ajhg.2017.04.015 28552198 PMC5473734

[36] Bouilly J Messina A Papadakis G Cassatella D Xu C Acierno JS. DCC/NTN1 complex mutations in patients with congenital hypogonadotropic hypogonadism impair GnRH neuron development. Hum Mol Genet (2018) 27(2):359–72. doi: 10.1093/hmg/ddx408 29202173

[37] Xu C Messina A Somm E Miraoui H Kinnunen T Acierno JC. KLB, encoding beta-klotho, is mutated in patients with congenital hypogonadotropic hypogonadism. EMBO Mol Med (2017) 9(10):1379–97. doi: 10.15252/emmm.201607376 PMC5623842 28754744

[38] Pitteloud N Hayes FJ Dwyer A Boepple PA Lee H Crowley WF Jr. Predictors of outcome of long-term GnRH therapy in men with idiopathic hypogonadotropic hypogonadism J Clin Endocrinol Metab (2002) 87(9)4128–36doi: 10.1210/jc.2002-020518 12213860

[39] Renault CH Aksglaede L Wojdemann D Hansen AB Jensen RB Juul A. Minipuberty of human infancy - a window of opportunity to evaluate hypogonadism and differences of sex development? Ann Pediatr Endocrinol Metab (2020) 25(2):84–91. doi: 10.6065/apem.2040094.047 32615687 PMC7336259

[40] Fiorino A Manenti G Gamba B Bucci G De Cecco L Sardella M. Retina-derived POU domain factor 1 coordinates expression of genes relevant to renal and neuronal development. Int J Biochem Cell Biol (2016) 78:162–72. doi: 10.1016/j.biocel.2016.07.013 27425396

[41] Pereira JH Kim SH. Structure of human brn-5 transcription factor in complex with CRH gene promoter. J Struct Biol (2009) 167(2):159–65. doi: 10.1016/j.jsb.2009.05.003 19450691

[42] Haddad Y Adam V Heger Z. Ten quick tips for homology modeling of high-resolution protein 3D structures. PloS Comput Biol (2020) 16(4):e1007449. doi: 10.1371/journal.pcbi.1007449 32240155 PMC7117658

[43] Hutchins BI Kotan LD Taylor-Burds C Ozkan Y Cheng PJ Gurbuz F. CCDC141 mutation identified in anosmic hypogonadotropic hypogonadism (Kallmann syndrome) alters GnRH neuronal migration. Endocrinology (2016) 157(5):1956–66. doi: 10.1210/en.2015-1846 PMC4870868 27014940

[44] Turan I Hutchins BI Hacihamdioglu B Kotan LD Gurbuz F Ulubay A. CCDC141 mutations in idiopathic hypogonadotropic hypogonadism. J Clin Endocrinol Metab (2017) 102(6):1816–25. doi: 10.1210/jc.2016-3391 PMC5470764 28324054

[45] Kioussi C Carriere C Rosenfeld MG. A model for the development of the hypothalamic-pituitary axis: transcribing the hypophysis. Mech Dev (1999) 81(1-2):23–35. doi: 10.1016/s0925-4773(98)00229-9 10330482

[46] Turton JP Reynaud R Mehta A Torpiano J Saveanu A Woods KS. Novel mutations within the POU1F1 gene associated with variable combined pituitary hormone deficiency. J Clin Endocrinol Metab (2005) 90(8):4762–70. doi: 10.1210/jc.2005-0570 15928241

[47] Duittoz AH Forni PE Giacobini P Golan M Mollard P Negrón AL. Development of the gonadotropin-releasing hormone system. J Neuroendocrinol (2021) 34(5):e13087. doi: 10.1111/jne.13087 PMC9286803 35067985

[48] Simonian SX Herbison AE. Regulation of gonadotropin-releasing hormone (GnRH) gene expression during GnRH neuron migration in the mouse. Neuroendocrinology (2001) 73(3):149–56. doi: 10.1159/000054631 11307033

[49] Kramer PR Wray S. Novel gene expressed in nasal region influences outgrowth of olfactory axons and migration of luteinizing hormone-releasing hormone (LHRH) neurons. Genes Dev (2000) 14(14):1824–34. PMC316793 10898796

[50] Givens ML Rave-Harel N Goonewardena VD Kurotani R Berdy SE Swan CH. Developmental regulation of gonadotropin-releasing hormone gene expression by the MSX and DLX homeodomain protein families. J Biol Chem (2005) 280(19):19156–65. doi: 10.1074/jbc.M502004200 PMC2932481 15743757

[51] Herbison AE Porteous R Pape J-R Mora JM Hurst PR. Gonadotropin-releasing hormone neuron requirements for puberty, ovulation and fertility. Endocrinology (2008) 149(2):597–604. doi: 10.1210/en.2007-1139 18006629 PMC6101186

[52] King JC. Rubin BS dynamic alterations in luteinizing hormone-releasing hormone (LHRH) neuronal cell bodies and terminals of adult rat. Cell Mol Neurobiol (1995) 15(1):89–106. doi: 10.1007/BF02069560 7648612 PMC11563131

[53] Clarkson J Han SY Pie R McLennan T Kane GM Ng J. Definition of the hypothalamic GnRH pulse generator in mice. Proc Natl Acad Sci USA (2017) 114(47):E10216–23. doi: 10.1073/pnas.1713897114 PMC5703322 29109258

[54] Nagae M Uenoyama Y Okamoto S Tsuchida H Ikegami K Goto T. Direct evidence that KNDy neurons maintain gonadotropin pulses and folliculogenesis as the GnRH pulse generator. Proc Natl Acad Sci USA (2021) 118(5):e2009156118. doi: 10.1073/pnas.2009156118 33500349 PMC7865162

[55] Campbell JN Macosko EZ Fenselau H Pers TH Lyubetskaya A Tenen D. A molecular census of arcuate hypothalamus and median eminence cell types. Nat Neurosci (2017) 20(3):484–96. doi: 10.1038/nn.4495 PMC5323293 28166221

[56] Sanz E Quintana A Deem D Steiner RA Palmiter RD McKnight GS. Fertility-regulating Kiss1 neurons arise from hypothalamic POMC-expressing progenitors. J Neurosci (2015) 35(14):5549–56. doi: 10.1523/JNEUROSCI.3614-14.2015 PMC4388920 25855171

[57] Huisman C Cho H Brock O Lim SJ Youn SM Park Y. Single cell transcriptome analysis of developing arcuate nucleus neurons uncovers their key developmental regulators. Nat Commun (2019) 10(1):3696. doi: 10.1038/s41467-019-11667-y 31420539 PMC6697706

