# Supplementary material for: POU6F2 mutation in humans with pubertal failure alters GnRH transcript expression
Source: Front Endocrinol (Lausanne). 2023 Aug 1;14:1203542. doi: 10.3389/fendo.2023.1203542 (PMC10436210; doi:10.3389/fendo.2023.1203542)
Supplement: Supplementary file 1 [file Table_1.docx]

|  | ClinGen Allele Registry | dbSNP | Canonical  NM_001370959.1 | ClinVar | ClinVar Accession#: |
| --- | --- | --- | --- | --- | --- |
| p.Gly601Arg  c.1801G>A | CA4227923 | [rs775692137](http://www.ncbi.nlm.nih.gov/snp/775692137) | c.1888G>A p.Gly630Arg | SUB13070478 | SCV003915726 |
| p.Asn629His  c.1885A>C | CA4227932 | [rs145961110](http://www.ncbi.nlm.nih.gov/snp/145961110) | c.1972A>C p.Asn658His | SUB13071712 | SCV003915743 |
| p.Pro74Leu  c.221C>T | CA4227448 | [rs373840004](http://www.ncbi.nlm.nih.gov/snp/373840004) | c.308C>T p.Pro103Leu | SUB13071423 | SCV003915738 |
| p.Gly92Glu  c.275G>A | CA157629906 | [rs940346614](http://www.ncbi.nlm.nih.gov/snp/940346614) | c.362G>A p.Gly121Glu | SUB13071164 | SCV003915729 |
| p.Pro287Arg  c.860C>A |  |  | c.947C>A p.Pro316Gln | SUB13071958 | SCV003915836 |
| p.Val336Leu  c.1006G>C | CA4227710 | [rs778523090](http://www.ncbi.nlm.nih.gov/snp/778523090) | c.1093G>C p.Val365Leu |  |  |
| p.Pro408Leu  c.1223C>T | CA367304356 | rs1226845805 | c.1310C>T p.Pro437Leu |  |  |
| p.Arg494Trp  c.1480C>T | CA4227844 | [rs767635644](http://www.ncbi.nlm.nih.gov/snp/767635644) | c.1567C>T p.Arg523Trp |  |  |
| p.Asn118Ser  c.353A>G |  |  | c.440A>G p.Asn147Ser |  |  |
| p.Ser264Ala  c.790T>G | CA4227642 | [rs760233765](http://www.ncbi.nlm.nih.gov/snp/760233765) | c.877T>G p.Ser293Ala | 2205616 |  |
| p.Ser264Ala  c.791C>A | CA4227643 | [rs765896780](http://www.ncbi.nlm.nih.gov/snp/765896780) | c.878C>A p.Ser293Tyr | 2205617 |  |
| p.Arg445Trp  c.1333C>T | CA4227804 | [rs540890506](http://www.ncbi.nlm.nih.gov/snp/540890506) | c.1420C>T p.Arg474Trp |  |  |
