# Supplementary figures and images for: POU6F2 mutation in humans with pubertal failure alters GnRH transcript expression
Source: Front Endocrinol (Lausanne). 2023 Aug 1;14:1203542. doi: 10.3389/fendo.2023.1203542 (PMC10436210; doi:10.3389/fendo.2023.1203542)

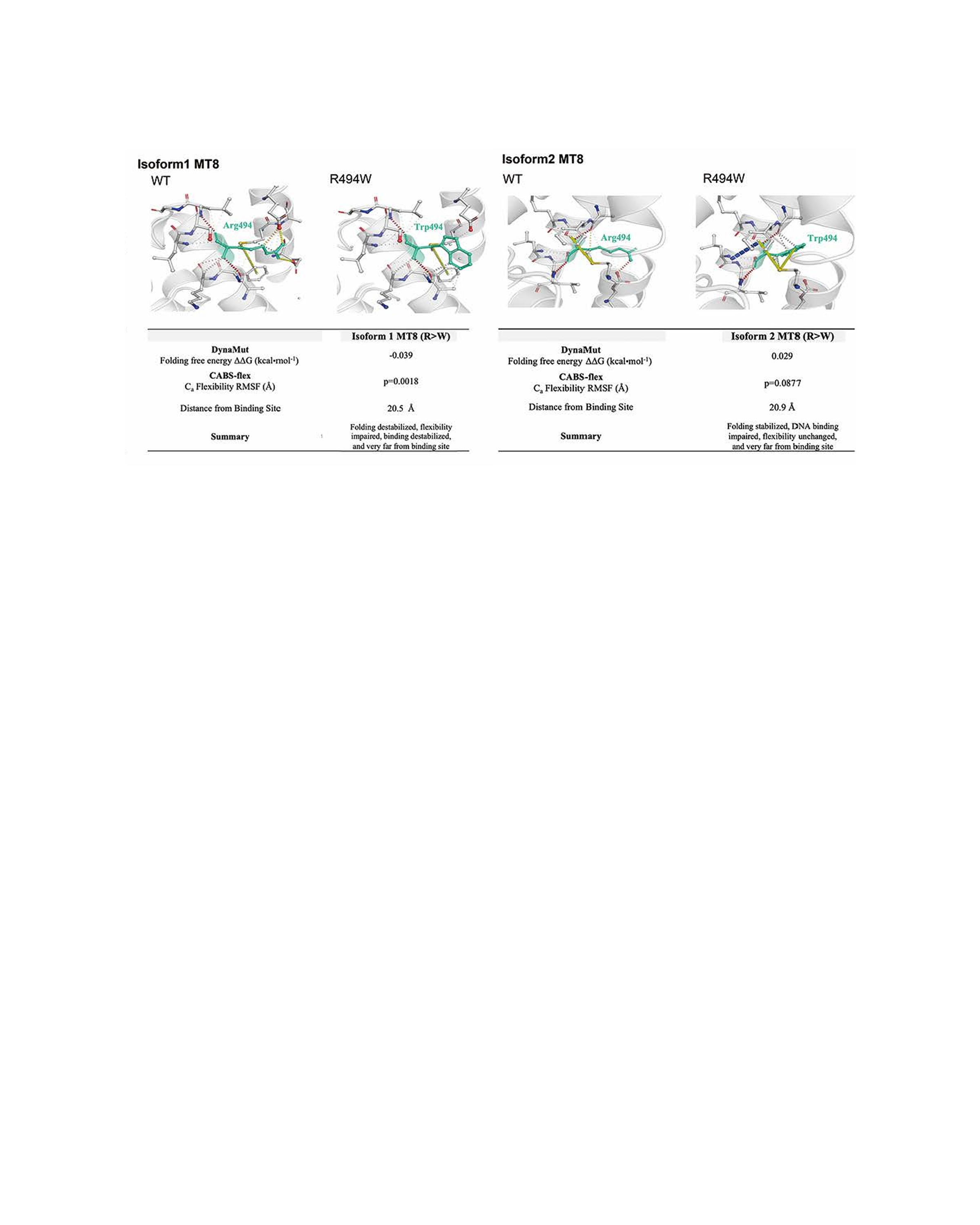

Supplement: Supplementary Figure 1 — In silico analysis of MT8. [file Image_1.jpeg]

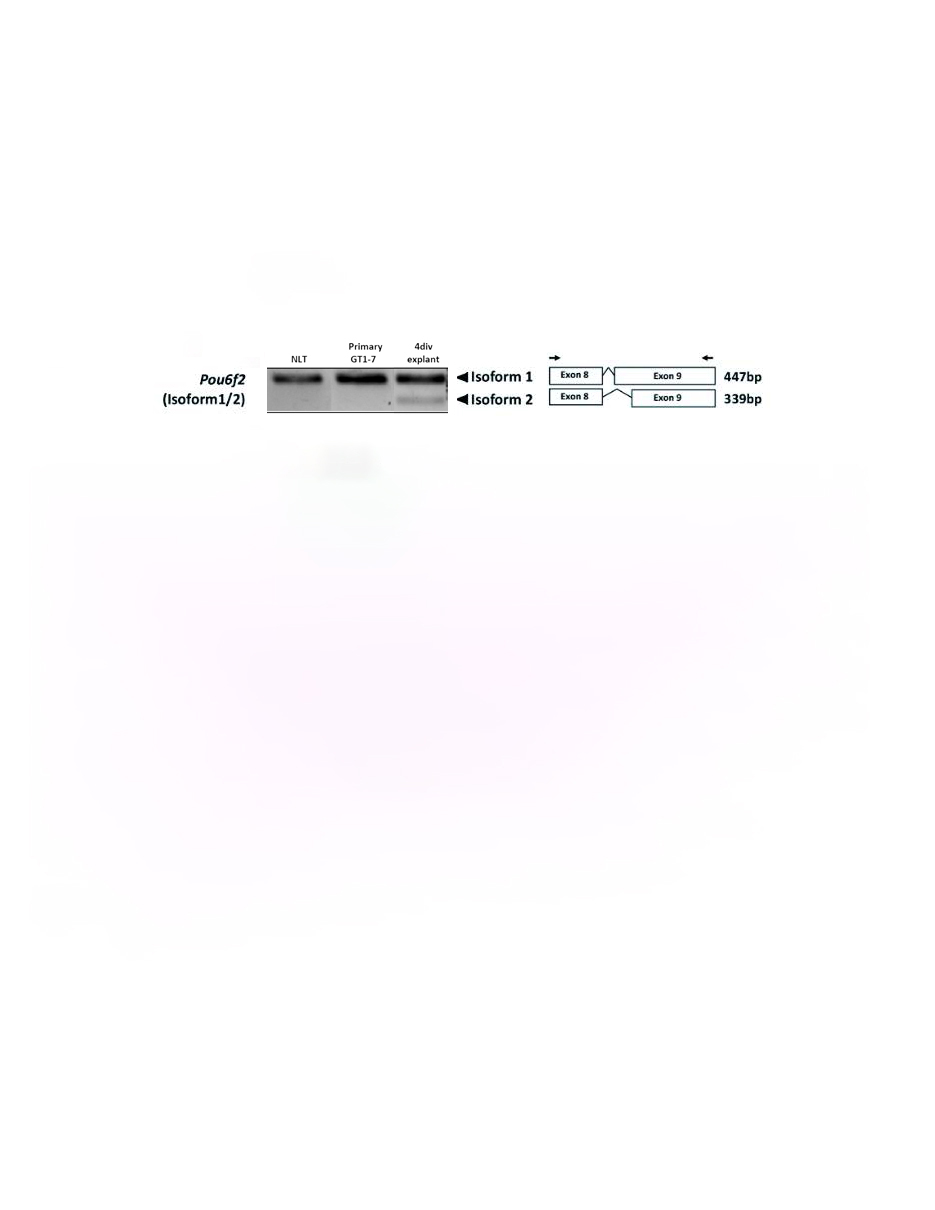

Supplement: Supplementary Figure 2 — Gel image of RT-PCR analysis performed on two GnRH mouse cells lines (18, 19). Top band (447 bp) shows isoform1 and bottom band (339bp) shows isoform2 which is skipping 108bp by alternative splicing on exon 9. Both cell lines expressed only isoform1. 4div explant= nasal explant (16, 17) was used as a positive control. [file Image_2.jpeg]
